# Development of a military mental health training program aiming to promote mental health and operational readiness in the Danish armed forces: an intervention mapping approach

**DOI:** 10.3389/fpubh.2025.1676193

**Published:** 2025-11-19

**Authors:** Anders Kjærgaard, Karen-Inge Karstoft, Bjarke Wæhrens Schmidt, Jeanette Wassar Kirk

**Affiliations:** 1 Danish Veterans Centre, Department of Military Psychology, Karup, Denmark; 2 Department of Psychology, University of Copenhagen, Copenhagen, Denmark; 3 Danish Veterans Centre, Research and Knowledge Centre, Ringsted, Denmark; 4 Department of Clinical Research, Hvidovre University Hospital, Hvidovre, Denmark; 5 Department of Health and Social Context, National Institute of Public Health, University of Southern Denmark, Copenhagen, Denmark

**Keywords:** mental health literacy, mental health training, operational stress, operational readiness, intervention mapping

## Abstract

Mental health and operational readiness are essential to soldiers’ ability to perform under pressure. Yet within military systems, the mental domain has often been overlooked or insufficiently integrated into formal training structures. Although international programs have aimed to enhance mental health literacy and resilience among service members, evidence regarding their applicability across national contexts remains limited. This is the first comprehensive mental health training program that has been developed or evaluated in Denmark. This paper addresses that gap by outlining the development of *Military Mental Training*, the first tailored program designed to promote mental health and psychological stress management skills and long-term operational effectiveness among personnel. We used the Intervention Mapping framework to construct the program. This involved a systematic six-step process, including stakeholder input, needs assessment, performance and change objectives, and evidence-based components such as stress management techniques and psychoeducation components about stress, mental health and coping. The program offers a contextually adapted, theory-driven approach to strengthening soldiers’ mental health literacy and coping capacities from the earliest stages of military training that move beyond implicit assumptions and toward structured, culturally adapted and theory-informed approaches. As such, this paper offers a replicable roadmap for other Defense organizations seeking to embed psychological readiness more explicitly into core training structures. However, further research is needed to assess results of the program in terms of acceptability, feasibility, and long-term effectiveness in real-world military settings. Beyond its military application, the training provides soldiers with transferable skills for managing stress in both service and civilian life.

## 1 Introduction

Amid ongoing geopolitical instability, military personnel are regularly deployed to high-demand environments, increasing their exposure to psychological risks. In Denmark, approximately 10% of veterans from high-intensity missions such as those in Iraq and Afghanistan were predicted to experience symptoms of severe depression or PTSD post-deployment, consistent with international estimates (2, 3, 75). The prevalence of such conditions is closely linked to the mission context and threat environment; however, their impact on individuals, families, and society is substantial and far-reaching, contributing to long-term health burdens and diminished workforce capacity (4–6). Moreover, soldiers face a range of sustained pressures even during peacetime duties, while navigating a volatile, uncertain, complex, and ambiguous (VUCA) environment (7). These demands highlight the need for preventative mental health initiatives that strengthen day-to-day coping and resilience beyond clinical treatment (1, 8, 9). In response, the Danish Ministry of Defense tasked the Danish Veterans Centre with developing a training program to reduce psychological strain among personnel (10). This initiative reflects a broader trend in research on military mental health interventions, where increasing attention has been given to the design and evaluation of structured training programs.

According to recent research, well-designed and context-specific mental health training programs can help prevent or mitigate psychological problems if they are properly developed, tested, and implemented (11–13). Such programs share the goal of enhancing mental health literacy - defined as individuals’ knowledge, attitudes, and beliefs about mental health (14, 15)—as well as mental readiness (11). Castro and Adler (16) argue that introducing mental health and stress management training during basic training may normalize mental health topics and ensure that soldiers acquire stress management competencies alongside core military skills (11, 17, 18). To be effective, however, training must be embedded within the broader military structure and occur through daily routines across the entire deployment cycle to reinforce skill development and sustain impact (11, 19). However, despite such promising evidence, translating training programs into practice presents several challenges that can undermine their effectiveness.

A general challenge in the implementation of mental health training programs is the inconsistent use of different theoretical concepts and definitions. Research has indicated that this inconsistency can create confusion about the intended target of the intervention, how it is implemented, and how outcomes are assessed and evaluated (20–22). Slep et al. (23) further note that many evidence-based programs encounter difficulties or diminish in efficacy over time due to weak or inadequate implementation strategies, insufficient organizational support, and poor adaptability to evolving institutional priorities. Insight from implementation science indicates that high complexity in training program design can hinder both organizational change and adaptation within target groups (24). Common barriers in the military include a lack of support or “buy-in” from local leadership, logistical challenges, and insufficient sustainable resources (11, 23, 25). It is therefore essential that the surrounding environment and organizational culture are aligned with, and capable of reinforcing the intended outcomes (26, 27, 71). Polusny and Erbes (28) suggest that integrating implementation principles with an emphasis on military culture, structured planning frameworks and outcomes offers a promising pathway for developing interventions that are both effective and sustainable. This reflects a growing scientific interest in not only what works, but in understanding how programs can be adapted into a specific context (69). To obtain this, methodological frameworks must be context-adaptable, not only addressing barriers but also aligning with the specific demands and values of a military culture (72). To address these challenges in a structured manner, researchers have increasingly turned to methodological frameworks that guide both development and implementation of complex health interventions.

One such framework is provided by the United Kingdom Medical Research Council (MRC), whose structured guidance for developing and evaluating health interventions is widely recognized and adaptable to military contexts (29, 30). Another is Intervention Mapping [IM; (26)] which offers a systematic, step-by-step process for designing complex behavioral interventions with a strong emphasis on cultural and contextual relevance. Together, these frameworks are complementary. While the MRC framework ensures methodological rigor and staged evaluation, IM enhances the practical tailoring of interventions to specific organizational cultures such as those found in military settings, thereby strengthening both implementation quality and long-term effectiveness (26). IM consists of six iterative steps: (1) Logic Model of the Problem, (2) Program Outcomes and Objectives, (3) Program Design, (4) Program Production, (5) Program Implementation Plan, and (6) Evaluation Plan (26). These frameworks are not only theoretical but have already been applied in several military contexts, offering insights into their practical use.

Three recent studies have used IM in developing performance-enhancement programs for military populations in Canada (31), Belgium (32), and the UK (33), respectively. While there were differences in both samples and approaches across these studies, they all focused primarily on elite or specialized military units. Studies embracing the entire Defense, including basic-level training have employed alternative approaches [e.g., (12, 13, 34–36)]. These findings identified a knowledge gap in research and underscore the need for a thorough integration of implementation science into military mental health programming, with particular attention to uptake and long-term sustainment (11, 17, 37). Against this backdrop, the current project was designed to contribute to the field by systematically developing and specifying a context-adapted program for Danish soldiers.

The objectives of this study were as follows: (1) systematically develop a contextually adapted mental health training program for Danish soldiers that can serve as a foundation for future evaluation of feasibility, acceptability, and effectiveness and (2) identify and operationalize key program outcomes and change objectives, defined by Bartholomew Eldredge et al. (26) as concrete elements that describe what needs to change in order to achieve the desired intervention outcomes, as well to guide the design and implementation of the program.

## 2 Methods

This study applied the IM framework (26) to guide the development of a mental health training program tailored for Danish soldiers. The six IM steps with respective subdomains structured the entire development process during which data analysis was performed in Step 1, followed by iterative co-creation with relevant stakeholders in Steps 2–5, and evaluation planning in Step 6.

### 2.1 Step 1

Establishing a planning group. Within the constraints of internal resource allocation, the initial project team consisted of two military psychologists and an army officer from the Danish Veterans Centre. Members were identified by the Chief of Department of Military Psychology at the Veterans Centre. The group was later expanded to include two additional military psychologists. Needs assessment. The needs assessment was informed by three primary sources: (1) literature reviews, (2) ongoing meetings with relevant key stakeholders, and (3) a user survey. Data from these sources collectively informed the identification of relevant behavioral targets, environmental conditions, and organizational factors that would shape the development and subsequent implementation strategy of the training program.

#### 2.1.1 Literature searches

As part of the initial development phase, three literature searches were conducted in early 2021 to identify evidence and the current state of existing military mental health training programs and their key components. Search strings can be found under supplementary material.

#### 2.1.2 Stakeholder engagement

During the development phase 2021–2024, several initiatives to gather information were launched, including focus group interviews: workshops, networks and forums for dialogue, ensuring broad representation (38). For example, in the beginning of 2021, an ongoing dialogue was established with a representative from the Medical & Health Command, responsible for, among other things, military physical training. The first outcome of this dialogue was a co-production of a user survey that was carried out mid-2021. In addition, meetings were held with international partners from, among others, the United States, Canada, Netherlands, and Norway with the aim of sharing exchanging experiences. In the beginning of 2022, a meeting and a focus group interview were held with the respective Branch Sergeant Majors and Non-Commissioned Officers (*N* = 4) to present ideas, engage in discussions, and obtain support so that they could later serve as structural ambassadors (implementation enablers) in their respective branches of the armed forces. Mid 2022, another key meeting was a workshop held with representatives (*N* = 10) from various structured education programs and the Defense Academy—all relevant implementation enablers for maintaining an ongoing buy-in during program implementation within their organizational structure. Later, a meeting was held with the conscription council, which represents all conscripts, with the aim of informing about the program and securing support from this level.

#### 2.1.3 User survey

A survey was carried out in collaboration with the Medical & Health Command in the Spring of 2021 intended to capture end-users’ current experiences, attitudes, and motivation in relation to physical and mental training, including perceived barriers concerning mental health, mental training, stress management and aspects of implementation.

### 2.2 Step 2

To systematically identify and operationalize change objectives, established behavioral frameworks were selected based on the information gathered in Step 1. The outcomes objectives were then broken down into performance objectives specifying which concrete actions participants must take to meet that particular program objective.

To ensure the MMT program was theoretically robust and practically relevant, we chose to develop matrices with inspiration from Mattie et al. (31) which resulted in a series of matrices linking key performance objectives to their underlying behavioral determinants (73). For each performance objective, we identified relevant determinants of behavior, informed by empirical evidence, stakeholder input, and contextual knowledge of the Danish military environments gathered in Step 1.

Each determinant was then mapped to a corresponding domain in Theoretical Domains Framework [TDF; (39)], which provided a structured lens to capture a broad range of psychological and contextual factors influencing soldiers’ behavior (e.g., knowledge, social influences). This ensured conceptual clarity and theoretical alignment. Subsequently, we integrated the behavior change wheel [BCW; (40)], including its COM-B model, to guide intervention design. Whereas TDF helped identify *what* determinants mattered, BWC provided a systematic way to translate these into *how* change could be achieved.

For example, reinforcing adaptive coping skills (41) and motivating soldiers through leaders who model effective stress management (42) are strategies that align with BCW’s emphasis on building capability, opportunity, and motivation. These approaches aim to consolidate behaviors into lasting habits through repeated practice (43). For each determinant-domain pair, evidence-based behavior change techniques (BCT; (40)) were then selected and operationalized into practical strategies suitable for delivery in a Danish military training context.

In addition to TDF and BCW, key perspectives from Self-Determination Theory (SDT; (44)) were integrated to ensure that the intervention not only targeted performance but also supported the basic psychological needs for autonomy, competence, and relatedness. SDT is particularly relevant in a military context, where hierarchical structures and operational demands can risk undermining intrinsic motivation if training is perceived as purely instrumental. Embedding SDT elements therefore helped ensure that the program balanced organizational goals with individual motivation and wellbeing.

### 2.3 Step 3

Each component of the program was evaluated in terms of its potential contribution to operational effectiveness, ensuring that the program remained feasible to implement and strategically aligned with the demands of military readiness. Subsequent pilot tests (Step 4) fine-tuned the final design of the various programs, MMT level 1–3. *Theoretical foundations.* Behaviorally oriented theories commonly applied in health promotion were explored to guide the development process. In particular, the training program was designed to align with fundamental motivational processes where SDT provides a central theoretical foundation (44). SDT offers a robust framework for understanding how intrinsic motivation, autonomy, competence, and relatedness can be important drivers within the context of military training. While the program content primarily focuses on strengthening and developing individual competencies and self-efficacy, each session concludes by linking these skills as relevant to the broader group context. As part of Step 3, instructional techniques and key program components were selected to address the specific change objectives identified at the individual level (26).

Knowledge and psychoeducation constitute a starting point in most mental health and resilience-building programs (11, 16, 17, 37, 45). However, to ensure meaningful engagement and buy-in, soldiers must first understand the clear rationale for the program’s relevance—addressing the question of “what’s in it for me?” To promote buy-in, each lesson includes the purpose, quotes and videos from relevant and relatable figures, including special forces who serve as role models for many, as well as former participants who share how they have applied the content in their respective contexts. Moreover, lessons are supplemented with empirical data and operational insights from the Veterans Centre, highlighting the most frequently reported stressors experienced by Danish soldiers in recent years.

At the Danish Veterans Centre, mental health literacy has been shaped by an adapted “Stress Continuum Model” (46) used as a self-assessment tool for symptoms and guidance on finding the right help (46, 47). Integrated stress management skills include evidence-based coping strategies to enhance performance and well-being (48, 49). Self-regulation plays a key factor in strengthening soldiers’ stress management skills and overall health. The capacity to adaptively manage one’s thoughts, emotions, and behaviors in a flexible manner is considered pivotal in why some individuals adjust more effectively when faced with demanding or high-stress environments (28). To ensure alignment with existing best practices and maintain contextual relevance, selected and targeted skills were adopted primarily from the Canadian Armed Forces R2MR program and the principles recommended by Castro and Adler (16).

### 2.4 Step 4

The overall purpose of this step was to obtain a reality check on the developed program and answer the questions of where and how potential participants would interact with the program (26). We collaborated with the Defense Media Center to produce online content and coordinated with internal stakeholders to develop printed handouts aligned with the updated material. During this phase, detailed learning plans for the MMT curriculum were developed using the Danish armed forces’ standardized template. This template requires every subject to be articulated in terms of specific learning objectives aligned with the Danish Qualifications Framework for Lifelong Learning (50). That framework offers a structured, level-based taxonomy of all officially recognized Danish credentials from primary education through university degrees and continuing professional development and maps each national level to its counterpart in the European Qualifications Framework (EQF).

#### 2.4.1 Pilot testing

To obtain feedback on the program’s relevance and applicability across subcultures within the Danish armed forces a series of pilot testing took place in the period 2022–2024. The online questionnaire was based on the theoretical framework of acceptability [TFA; (51)] covering nine central questions. The TFA provides a validated, theory-based structure for measuring intervention acceptability across key dimensions, making it well-suited for structured surveys. Currently, as a standard, all participants complete an online questionnaire designed to assess the program’s overall relevance and acceptability (51). Afterwards, participants provide verbal feedback on content, structure, and suggestions for improvement, thus supporting an ongoing acceptability study. The pilot testing included participants from all branches of the armed forces, and with participants varying in age and ranks. In August 2024 the first trainer-delivery to Air Force conscripts (level 1) was conducted. A fidelity check-in form of a customized checklist translated from the Canadian R2MR program was managed by a social worker, employed at the Veterans Centre. She had also received the MMT2 trainer program; and was instructed to evaluate the training program during the actual delivery. At the same time a military psychologist taught a similar group in the same material. Evaluations from the groups were compared.

### 2.5 Step 5

This step took place during 2021 alongside Steps 1 and 2 during which identification of relevant key stakeholders and clarification of roles were discussed and prioritized. *Adopters* refer to those who formally decide to integrate the program into existing training systems or curricula; *implementers* are the personnel operating/delivering the program in practice; and *maintainers* are those responsible for institutionalization and embedding the program into long-term structures and routines to support its ongoing life (24). *Champions,* individuals who actively promote and sustain enthusiasm for the program (52), were recognized as critical actors within the latter two groups. This approach also drew on the Behavior Change Wheel framework, emphasizing the importance of enabling policies across seven categories to support successful implementation (40). Attention was given to the risk of training effects diminishing over time and efforts were made to communicate the need for periodic updates to both program content and delivery methods when opportunities occurred.

### 2.6 Step 6

The formulation of an evaluation plan marks the final step in the IM process. A key question during this phase was determining when the development phase ends and evaluation begins.

## 3 Results

### 3.1 Step 1: logic model of the problem

#### 3.1.1 Establishing a planning group

Although not involving all branches of the Defense, the planning group had accumulated operational knowledge across all service branches and was therefore expected to be able to provide broad representation across the organization. The initial strategy, understood as a plan that integrates major goals, policies, and action sequences into a cohesive whole (53), was formulated as: “direction over speed.” A bottom-up, iterative approach that prioritized and emphasized gradual development and early data collection from the pilot tests was used to assess the intervention’s acceptability.

#### 3.1.2 Literature reviews

The first review focused on “mental skills training in the armed forces” and was based on a review that had recently been published (49). Inconsistencies in the clarity of information provided were observed among the studies examined. While some studies offered a comprehensive breakdown of the mental skills training program, including its applications and associated activities, others provided only a cursory outline with ambiguous descriptions of the specific instructions or applications of these skills. Notably, a significant discrepancy was observed in the amount of time dedicated to mental skills training and application within the overall program (49).

The second review examined the “effects of mental training programs in military contexts.” The rationale for this was to identify whether any assessment measure would stand out for recommendation in a military context. In summary, there was great variance among the methods used to assess program effects across the 15 selected studies, which was also noted in the systematic review by Harden et al. (74). A gold standard for evaluating comprehensive mental training programs in military contexts could not be identified in the literature search. This gap was further confirmed by leading experts within the field (i.e., Amy Adler and Suzanne Bailey) in a personal correspondence December 5, 2024. Therefore, a clear recommendation could not be made regarding which methods should be included to optimally measure effects.

The third review examined delivery methods, with particular emphasis on the “train the trainer” approach. The rationale for exploring this topic further was based on experiences from the Canadian R2MR-concept, where fidelity issues (where trainers drifted and deviated from the manuals) had played a significant role in their train the trainer-program (54). In summary, there was great variance in the selected studies regarding the effect of the train-the-trainer method because of differences in how the effect was measured. It is therefore difficult to evaluate an unambiguous effect of training since it is not clear why some studies found a positive effect whereas other studies found a lower effect of their train the trainer method compared to alternatives. However, despite the lack of evidence, the method is consistent with the prevailing training method (best practice) in the Danish armed forces as well as other NATO countries. A focus point consequently centered on the formulation of the teaching material so that “the average trainer” would understand it (Step 4).

#### 3.1.3 Stakeholder engagement

As a result of the ongoing dialogues with different stakeholders, a formal Human Performance Optimization (HPO) network was established under the Defense Academy, providing a platform for interested stakeholders to continuously stay informed, as well as an opportunity for the planning group to obtain continuous feedback when presenting new ideas or results. This community would also facilitate ongoing program validation and support implementation buy-in. Annual meetings were established as the standard and aligned with other networks under the Defense Academy. Importantly, the HPO network functions as a mechanism for multilevel engagement: it enables soldiers and end-users to voice concerns and suggestions, allows middle managers and instructors to act as mediators and gatekeepers of implementation, and secures institutional endorsement through formalized alignment with the Defense Academy. In this way, stakeholder engagement becomes not only a means of information exchange but also a structured approach to securing buy-in across levels critical for sustainable implementation.

#### 3.1.4 User survey

A user survey yielded over 3,000 responses from active soldiers across all branches of the armed forces, representing more than 15% of the total active military population at the time (Veterans (55)). Key findings demonstrated a significant demand for enhanced mental skills training: 62% of respondents indicated a desire for more education and training in mental tools for stress management, while 17% were undecided. Barriers to practice were primarily attributed to “time pressure” and “general bustle” (44%) as well as “lack of competencies” (43%) (Veterans (55)). The survey results provided vital input for the development of the logic model, helping to identify behavioral and environmental determinants relevant to intervention development. Furthermore, these insights contributed to the formulation of strategic decisions regarding the design, delivery, and organizational positioning of the forthcoming mental health training program. The overall goals were stated as: (1) developing a program suitable and acceptable for a Danish military context and (2) demonstrate a positive effect on selected outcome parameters described further in Step 2. A detailed version of the initial logic model, which is a guide for program development and evaluation, is available online.

### 3.2 Step 2: program outcomes and objectives

Due to the broad implementation perspective across all branches of the Danish armed forces, it was necessary to define a set of sub-goals to guide development in a structured and feasible manner. Accordingly, and based on the collected data from Step 1, the planning group identified four key performance objectives: (1) increase awareness of the MMT program, (2) integrate MMT into structured military education curricula, (3) increase usage of stress-management skills in everyday routines and (4) educate trainers to deliver the MMT program with fidelity. These objectives served as a foundation for linking behavioral outcomes to specific change strategies and ensured alignment between program content, delivery mechanisms, and desired operational impact. Based on these performance objectives, two primary target populations were identified: (1) conscripts at basic training and (2) personnel engaged in a structured education, e.g., the Sergent- or Officer schools, to ensure that future leaders receive and accept the program, thereby enabling them to support the dissemination of the program and deliver it to new soldiers. In addition, strategic emphasis was placed on the permanent personnel who did not have access to the program through structured training. They were to be reached via open courses. The detailed matrices linking performance objectives for personnel to determinants and change objectives can be accessed online.

### 3.3 Step 3: program design

The selection of components for each level of MMT is shown in Table 1. Levels 2 and 3 retain the core curriculum and add on dedicated modules that prepare participants to teach and mentor others.

**Table 1:** Design of the various programs.

Design of the various programs	Level 1	Level 2	Level 3
	End users (basic training)	Trainers (schools)	Subject matter instructors
Duration	1 day (7,5 h)	2 days (16 h)	3 weeks (27 h)
Content	One theoretical module about stress in a Danish military context, mental health literacy and prevention initiatives including the stress-continuum model One theoretical module about mental training in general and mental training in military contexts with examples Four practical skill modules (goalsetting, self-talk, visualization, breathing techniques). Each module focuses on short-term use as well as long term use related to both individual and unit	One theoretical module about stress in a Danish military context, mental health literacy and prevention initiatives including the stress-continuum model One theoretical module about mental training in general and mental training in military contexts with examples Four practical skill modules (goalsetting, self-talk, visualization, breathing techniques). Each module focuses on short-term use as well as long term use related to both individual and unit One theoretical module about implementation science Six practical training modules regarding the mentioned topics	This course is a combined three-week program held in collaboration with the Medical & Health Command. It combines physical training, military unit training, and MMT training. The course comprises the two-day trainer package supplemented by lectures that elaborate on mental techniques and the principles of implementation science. In addition, participants engage in practical obstacle and training courses in which the MMT is integrated with both physical conditioning and unit-level exercises.
Prerequisites	No preparation.	Some preparation in the form of reading background material. Typically, this is for students at Sergeants schools or other relevant venues.	More preparation, e.g., - having passed another course - having read the MMT book for this course. - Typically, Drill Sergeants or experienced personnel
Delivery	Military psychologists/local /subject matter experts	Instructors are experienced military psychologists.	Experienced military psychologists/military physical trainers.

Based on the experiences from the pilot testing in Step 4, MMT1-3 now employs a standardized format to maintain feasibility and fidelity under typical military constraints, while embedding built-in options for individual customization to support autonomy. This dual approach allows service members to adapt the material in personally meaningful, contextually relevant ways, enhancing skill transfer across diverse operational environments.

#### 3.3.1 Size of training groups

The appropriate size of training groups was yet another key consideration in the program design. While different branches operate with varying unit sizes, the planning group decided that group should ideally consist of 12–25 participants to ensure effective group dynamics and interactions. However, recognizing the realities of military settings, it was acknowledged that units may occasionally face logistical constraints that necessitate larger groups.

#### 3.3.2 Delivery method—train the trainer

During Step 1, it was determined that the program would adopt a train-the-trainer model to ensure scalability across the Danish armed forces. This decision was informed by the Veterans Centre’s limited capacity for direct program delivery, and by evidence from comparable international programs (18, 37, 56). The train-the-trainer approach equips selected personnel to deliver the program to peers, fostering a self-sustaining learning culture within the organization (57, 58). This implementation strategy aligns with existing practices in the Danish armed forces and was thus considered both feasible and culturally congruent. The program content therefore had to be accessible and deliverable without clinical expertise, targeting the “average trainer” profile described by Van den Berge, (18). After completing the Level 2 trainer course, a support structure was introduced based on feedback from participants: monthly virtual voluntary peer-support sessions facilitated by military psychologists from the planning group. These sessions aim to offer technical support and peer exchange. In addition, trainers are also able to request one-on-one support between sessions. Over time, it is anticipated that experienced subject matter instructors will gradually take on this supportive role as their numbers increase.

#### 3.3.3 Delivery platforms

Based on comments from stakeholders, the planning group determined that the primary format for the program should be face-to-face, to support engagement, interaction, and contextual relevance. To enhance accessibility and reinforce key messages, selected content was also made available through online platforms. Concurrently, the planning group emphasized the importance of maintaining consistency across units and cautioned against the proliferation of locally adapted versions which could compromise fidelity and hinder evaluation efforts. During a 2018 study visit to Canada, co-facilitation (where two instructors deliver the training jointly) was identified as a promising method for improving instructional quality, managing group dynamics and adopt locally (54). However, due to current resource constraints, widespread implementation of this model was deemed unfeasible. Instead, trainers were encouraged to actively involve participants and draw on their lived experiences to place the content within local context and thus strengthen relevance within their specific operational environments.

### 3.4 Step 4: program production

Materials produced in this step included: presentation slides to support instruction; an action card describing four stress regulation strategies for use before, during, and after a task (target group: Levels 1–3); a revised version of the stress continuum leaflet with integrated stress management skills (Levels 1–3); a trainer’s handbook (Level 2); a foundational manual for the MMT program (Level 3). Selected materials were made available on the Veterans Centre’s public website,^1^ while other resources were uploaded to internal course platforms used by the Danish armed forces.

#### 3.4.1 Pilot testing

The test period, and thus the development phase for MMT1 and MMT2, lasted until August 2024, when final programs were determined based on the feedback. The pilot tests for MMT3 were in November 2023 and November 2024. Results are shown in Table 2.

**Table 2:** Results from the pilot testing.

Time	N	1	2	3	4	5
		*n* (%)	*n* (%)	*n* (%)	*n* (%)	*n* (%)
How satisfied were you with the MMT program? ~ very unsatisfied [1] ↔ very satisfied [5]						
MMT1	66	0 (0%)	0 (0%)	3 (4.5%)	25 (37.9%)	38 (57.6%)
MMT2	147	1 (0.7%)	0 (0%)	6 (4.1%)	73 (49.7%)	67 (45.6%)
MMT3	32	0 (0%)	0 (0%)	1 (3.1%)	14 (43.8%)	17 (53.1%)
What did it take to participate in the MMT program? ~ requires no effort [1] ↔ requires a lot of effort [5]						
MMT1	66	0 (0%)	23 (34.8%)	9 (13.6%)	33 (50%)	1 (1.5%)
MMT2	147	4 (2.7%)	71 (48.3%)	8 (5.4%)	58 (39.5%)	6 (4.1%)
MMT3	32	0 (0%)	6 (18.8%)	3 (9.4%)	23 (71.9%)	0 (0%)
There are moral/ethical consequences when using MMT techniques? ~ strongly disagree [1]↔ strongly agree [5]						
MMT1	66	12 (18.2%)	22 (33.3%)	13 (19.7%)	16 (24.2%)	3 (4.5%)
MMT2	147	49 (33.3%)	55 (37.4%)	34 (23.1%)	7 (4.8%)	2 (1.4%)
MMT3	32	6 (18.8%)	15 (46.9%)	6 (18.8%)	0 (0%)	3 (9.4%)
Can MMT improve my performance as a soldier? ~ strongly disagree [1] ↔ strongly agree [5]						
MMT1	66	1 (1.5%)	0 (0%)	3 (4.5%)	21 (31.8%)	41 (62.1%)
MMT2	147	0 (0%)	1 (0.7%)	2 (1.4%)	56 (38.1%)	88 (59.9%)
MMT3	32	0 (0%)	0 (0%)	0 (0%)	12 (37.5%)	20 (62.5%)
It is clear to me how MMT techniques can work in the military context? ~ strongly disagree [1]↔ strongly agree [5]						
MMT1	66	1 (1.5%)	0 (0%)	4 (6.1%)	33 (50%)	28 (42.4%)
MMT2	147	0 (0%)	4 (2.7%)	7 (4.8%)	71 (48.3%)	65 (44.2%)
MMT3	32	0 (0%)	0 (0%)	0 (0%)	13 (40.6%)	19 (59.4%)
To what extent do you feel able to use the content from the MMT program? ~ very unsure [1]↔ very sure [5]						
MMT1	66	0 (0%)	5 (7.6%)	6 (9.1%)	44 (66.7%)	11 (16.7%)
MMT2	147	1 (0.7%)	4 (2.7%)	13 (8.8%)	96 (65.3%)	33 (22.4%)
MMT3	32	0 (0%)	3 (9.4%)	3 (9.4%)	19 (59.4%)	7 (21.9%)
Using MMT interfere with other priorities in my work as a soldier? ~ strongly disagree [1]↔ strongly agree [5]						
MMT1	66	18 (27.3%)	25 (37.9%)	18 (27.3%)	5 (7.6%)	0 (0%)
MMT2	147	19 (12.9%)	90 (61.2%)	20 (13.6%)	16 (10.9%)	2 (1.4%)
MMT3	32	5 (15.6%)	18 (56.2%)	6 (18.8%)	3 (9.4%)	0 (0%)
How acceptable is the MMT program overall to you? ~ completely unacceptable [1] ↔ completely acceptable [5]						
MMT1	66	0 (0%)	0 (0%)	6 (9.1%)	24 (36.4%)	36 (54.5%)
MMT2	147	1 (0.7%)	1 (0.7%)	2 (1.4%)	79 (53.7%)	64 (43.5%)
MMT3	32	0 (0%)	0 (0%)	2 (6.2%)	14 (43.8%)	16 (50%)
Overall, how satisfied have you been with the instructors? ~ very unsatisfied [1]↔ very satisfied [5]						
MMT1	66	0 (0%)	0 (0%)	2 (3%)	29 (43.9%)	35 (53%)
MMT2	147	0 (0%)	0 (0%)	4 (2.7%)	55 (37.4%)	88 (59.9%)
MMT3	32	0 (0%)	0 (0%)	1 (3.1%)	11 (34.4%)	62.5%)

The pilot results indicate that satisfaction and acceptability were consistently high across all three MMT courses, demonstrating a solid program content. Ethical concerns and practical interference were noted and remained relatively constant across all courses as issues that require special attention during delivery and later when integrated into everyday life.

### 3.5 Step 5: program implementation plan

Identification of relevant key stakeholders and clarification of roles were discussed with stakeholders and prioritized as presented in Table 3.

**Table 3:** The implementation ecology system – most relevant key stake holders.

Program users	Adopters	Implementers	Maintainers
- Recruits on basic training - Permanent personnel - Sergeants - Officers	- Veterans Centre - Academy of Defense - Army, Navy, Airforce commanders - Branch Sergeant Majors - Commanders at respective schools - Local commanders - The top leadership of the Danish Armed Forces	- Trainers - Subject matter experts - Unit-level leaders - Local commanders - Program champions	- Planning group - Trainers - Subject matter experts - Branch Sergeant Majors - Conscription council - Local commanders - Academy of Defense - Commanders at respective schools - Program champions

#### 3.5.1 Implementation outcomes

Based on the listed performance objectives (Step 2), the planning group decided to list two implementation outcomes as initially relevant: (1) acceptability (is the program well-received?) and (2) feasibility (can the program realistically be implemented in the specific context and everyday practice?) (59). Both outcomes were systematically assessed based on the feedback from the pilot testing and subsequently after each delivery through a combination of standardized questionnaires (self-report surveys) and participant responses (interviews with participants and focus group interviews). Data collection remains ongoing, and detailed findings will be reported in a subsequent publication.

#### 3.5.2 Performance objectives for implementation

As part of the implementation planning, *adoption,* defined as the formal decision by leadership or units to use the MMT program, was identified as a key implementation outcome as well as a vital performance objective (59). From the outset, full-scale adoption was considered contingent on institutional integration of the program into official learning plans and training descriptions. These documents define the core parameters for program delivery, including intended learning outcomes, instructional methods, duration, required materials, and logistical considerations. Accordingly, efforts were directed toward embedding MMT content into existing military educational structures to ensure sustained and consistent use across units. In addition to this system-level objective, the planning group outlined specific performance objectives tailored to relevant key stakeholders, including decision-makers, implementers, and end users. These objectives are summarized in Table 4 and were used to guide targeted implementation strategies.

**Table 4:** Performance objectives for implementation.

Role	Performance objectives
Adopters	- Approve the integration of MMT into existing education curricula. - Allocate sufficient resources (trainers, time) for the MMT rollout. - Communicate the strategic importance of MMT to all units. - Endorse the program visibly to create organizational legitimacy.
Implementers	- Complete required MMT trainer courses - Deliver MMT sessions following standardized manuals and protocols. - Integrate MMT content into unit training plans and routines. - Report delivery challenges and successes to program maintainers. - Collect and report participant feedback systematically (e.g., via surveys)
Users	- Attend and engage actively in MMT training sessions. - Practice stress management techniques pre, during, and post relevant everyday activities according to existing education curricula - - Provide honest feedback on training acceptability, relevance and applicability.
Maintainers	- Monitor the fidelity and quality of MMT delivery across units. - Provide mentorship and technical support to active trainers. - Update training materials based on feedback and evolving needs. - Ensure continuous institutional support for program sustainability. - Gather data on implementation outcomes to evaluate program success and justify continuation (e.g., via user surveys).

Embedding the mental health training into existing military education ensures consistency and alignment with institutional standards. Currently, a new structure for basic training is being developed in Denmark and will launch in 2026. The MMT program will then be mandatory as a stand-alone subject within in the first 3 months, with the aim of integration into other training modules. By integrating MMT across activities, the training becomes a visible and routine part of military service, increasing the likelihood that skills are retained and practiced.

### 3.6 Step 6: evaluation plan

The formulation of an evaluation plan marks the final step in the IM process. A key question during this phase is determining when development ends and evaluation begins. The planning group agreed that development could be considered complete when pilot feedback yields only minor or isolated suggestions which is in accordance with Malterud et al. (60) concept of information power. A primary evaluation criterion is acceptability, defined as the degree to which the target audience perceived the program relevant and engaging. Early indicators of effectiveness (e.g., increased knowledge, self-efficacy, or intent to change behavior) should be evident, even before full-scale outcome evaluation. Alignment with overarching strategic goals such as enhancing operational readiness and fostering a supportive command climate is also essential. Ongoing quality assurance and iterative refinement will remain necessary as broader effectiveness and scalability depend on continued evaluation, adaptation, and full integration into remaining education structures.

## 4 Discussion

The MMT program was developed using an IM approach with inspiration from previous work conducted in specialized military and security units by (31–33). Unlike these earlier efforts, the present program targets a broader segment of the armed forces, necessitating tailored design and delivery considerations. Challenges related to delivery and cultural fit emerged most clearly during Step 5 (implementation planning). Specifically, stakeholder coordination and logistical constraints highlighted the importance of allocating sufficient planning resources early in the process. In this project, delivery within a military culture required balancing top-down expectations with the practical realities of military operations and training rhythms. Negotiating the appropriate training length served as a practical example of Step 3, where theoretical design needed to align with contextual feasibility. This required compromise between logistical realities and professional judgment on the factors necessary to support meaningful behavior change. As shown in the Step 4 pilot results (Table 2), >90% of participants across levels rated the program acceptable and of practical relevance in their military context. Also, >90% thought the program could improve their performance as a soldier. However, a key focus point from the verbal feedback was to ensure that the program would not function as a “stand-alone” intervention. Instead, it should be intentionally structured for integration within existing curricula and training structures, supporting sustainability and institutional alignment. Both central goals of IM Step 5.

Leadership engagement also emerged as a critical implementation factor. Consistent with the roles identified in IM Step 5, the findings show that leadership support is particularly influential during early implementation, shaping unit norms and overall receptivity (61, 70). Conversely, when leaders fail to model commitment to mental health efforts, the potential for negative downstream effects increases (62, 63). These findings underscore the importance of equipping leaders not only with the training content itself, but also with tailored implementation guidance relevant to their influence and function. Importantly, securing sustainable buy-in must occur at multiple levels: at the individual level, soldiers need to perceive personal relevance and value (“what’s in it for me?”); at the meso-level, middle leaders and instructors act as gatekeepers who can either reinforce or hinder program uptake; and at the institutional level, organizational endorsement is required to align policies, resources, and expectations. Without coherent buy-in across these layers, implementation risks becoming fragmented and less effective.

The pilot tests in IM Step 4 showed that ethical aspects are important to consider. Results in Table 2 showed the greatest spread on this parameter indicating that implementing a mental health training program where operational performance is central raises ethical considerations balancing rearmament and operational demands while accommodating the psychological needs and motivational expectations of personnel. A potential risk is that mental health training becomes instrumentalized, used solely to optimize performance rather than to support the individual holistically (64, 65). To counter this, programs must clearly communicate a dual purpose: enhancing both operational readiness and personal wellbeing. Mandatory training also raises concerns about autonomy. Even within structured formats, programs must allow space for reflection and internalization. Personal reflections or self-assessments and other data use must be clearly bounded in terms of confidentiality and data use as fear of repercussions can inhibit openness. It must be explicit that training is educational, not evaluative and that no clinical judgments will be made.

Finally, leaders should be equipped not only with program content, but also with guidance on ethically responsible facilitation (62). Ethical reflection, cultural sensitivity, and clear role delineation are essential to ensure that such programs are not only effective and scalable, but also trusted, accepted, and sustainable. As the literature searches in Step 1 showed, these points can become blurred when adopting an entire program or parts of it, since validated components in one context may not automatically work in another (66, 67). This has implications for successful implementation, underscoring the need to adapt health programs to the specific organizational culture, define clear needs, and structure training as a developmental pipeline that builds skills progressively.

### 4.1 Limitations

While this paper aims to provide a transparent account of the development of MMT in the Danish armed forces, several limitations should be noted. First, the findings are context-specific and therefore its transferability to other contexts may be limited. For example, the composition of the planning group will have an impact on the ongoing process. The inclusion or exclusion of certain stakeholders may also have shaped priorities and emphasis in ways that could differ elsewhere. Thus, selection bias at the planning level should be considered when replicating the process. Second, the intervention- and implementation frameworks applied represent one possible approach. Alternative theoretical frameworks or evidence bases could have led to somewhat different program components or strategies. This limits direct generalization and underscores the importance of transparent reporting of the decision-making rationale, so that others can adapt the process to their own institutional context. Third, complexity and scale increase as the target population grows larger, and greater resources should be expected accordingly. Therefore, the current approach might not work as well in larger countries or systems with less centralized structures. Fourth, as the focus was primarily on intervention design, our conclusions are restricted to issues of feasibility and acceptability, rather than demonstrated effectiveness. Finally, the participatory approach may have introduced subjectivity in how needs and priorities were identified. While this approach enhances relevance and ownership, it also risks reflecting the perspectives of the most engaged participants rather than the full spectrum of potential stakeholders.

### 4.2 Future directions

Logistical tasks have been time consuming and need to be addressed in future resource planning. The next phase will involve a more thorough evaluation of the program’s feasibility and acceptability from the perspectives of both end-user and trainers. Empirical data will be gathered to assess the program’s effectiveness relative to its defined change objectives. The extent to which MMT is integrated into standard learning plans and institutional curricula as a fixed learning objective will be tracked longitudinally, and a follow-up user survey will assess program reach, engagement and perceived value within the current operational force. Finally, as noted by de Vries et al. (68) future research should track technological advances for monitoring soldiers’ health and readiness and, when feasible and operationally relevant, integrate biofeedback to enhance measurability and complement behavioral training.

## 5 Conclusion

This paper marks the first presentation of the IM approach to designing a mental health training program for an entire military population. In doing so, it offers a novel contribution by documenting development and implementation processes that are essential for replication and practical translation in other military contexts. IM was valuable in providing a structured alternative to intuitive program development that relies on assumed best practices or practitioner experience alone. The IM process ensured that behavioral goals, change methods, and delivery strategies were logically aligned and grounded in theory. This added transparency, coherence, and adaptability, all critical qualities in complex military settings. Still, cautions must be taken. Although there is no indication that mental training in general poses harm, it remains essential to rigorously evaluate its effectiveness before widespread implementation.

## Data Availability

The original contributions presented in the study are included in the article/Supplementary material, further inquiries can be directed to the corresponding author.

## Footnotes

^1^ www.veterancentret.dk/mmt
