# Supplementary material for: Development of a military mental health training program aiming to promote mental health and operational readiness in the Danish armed forces: an intervention mapping approach
Source: Front Public Health. 2025 Nov 19;13:1676193. doi: 10.3389/fpubh.2025.1676193 (PMC12673934; doi:10.3389/fpubh.2025.1676193)
Supplement: Supplementary file 1 [file Table_1.docx]

**
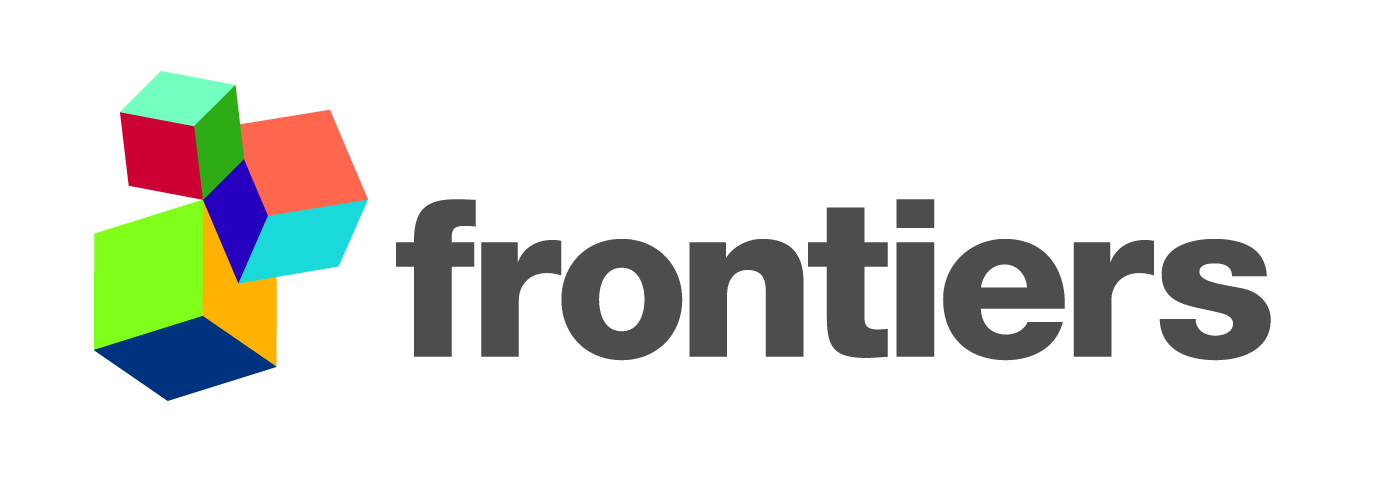
Development of a military mental health training program aiming to promote mental health and operational readiness in the Danish armed forces: A simple logic model**

Inspired by Strickland et al. 2019 (<https://www.mdpi.com/1660-4601/16/4/590>)



| **BASIC**  **ASSUMPTIONS** | **PRE-IMPLEMENTATION** | **PROPGRAM IMPLEMENTATION** | | | **PROGRAM EFFECTIVENESS** | | | |
| --- | --- | --- | --- | --- | --- | --- | --- | --- |
|  | **PROGRAM READINESS** | **INPUTS** | **ACTIVITIES/**  **MECHANISMS** | **OUTPUTS** | **SHORT TERM OUTCOMES** | **INTERMIDIATE OUTCOMES** | **LONG TERM OUTCOMES** |  |
| **MENTAL SKILL TRAINING**  *“The effectiveness of mental skills training (MST) in facilitating performance is well documented in the sport and performance psychology literature, with both formative (e.g. Greenspan & Feltz, 1989) and contemporary (e.g. Brown & Fletcher, 2017) meta-analyses demonstrating that psychological interventions are effective in enhancing human performance” (e.g. Mattie et al. 2020)*  **MENTAL HEALTH LITERACY**  *“Persistent stigma, lack of knowledge about mental health, and negative attitudes toward treatment are among the most significant barriers to military service members and veterans seeking behavioral healthcare” (e.g. Mohatt et al. 2017)*  **The purpose of the specific program is to improve basic mental health literacy and strengthen soldiers’ stress management skills in relation to be able to handle work demands better.**  A potential effect might be to mitigate psychological after-reactions post missions, which are costly on both a personal and societal level. | - Establishment of working group - Selected frameworks (MRC & Intervention mapping) as inspiration/planning frameworks - Hypotheses - Logic model of problem - Logic model of change - Reality check - Organizational knowledge and readiness (resources and commitment) - Involvement from relevant stakeholders - Planning process with relevant stakeholders (aim: to be enrolled in curriculums in structured educations) - **Program/intervention design/development** based on: - Literature reviews - NATO recommendations - Experiences from different national programs | - Different programs are developed (level I-III). - Materials are produces (leaflets, online, ect.) - Pilot studies to test and refine the developed programs (to identify barriers and facilitators in context) | - Continuing dialogues with relevant stakeholders (aim: to be enrolled in curriculums in structured educations) - Delivery of modified programs/interventions to selected groups (end users + trainers). | - Delivered interventions to end users in local workplaces (customized to Army, Navy and Air Force) - Awareness of the program spreads and interests increases (more courses). | - Trainers + end users are educated. - Training in selected groups (via trainers and/or military psychologists) - Program becomes mandatory at basic training level. - Program is integrated in important structured educations (basic training, sergeant + officer schools) | - Improved usage of stress management skills in selected groups (compared to baseline measures) - Improved Mental health literacy in selected groups (compared to baseline measures) | - Program is integrated in every structured education. - Improved usage of stress management skills (compared to baseline measures) - Improved Mental health literacy (compared to baseline measures) - Cultural change in how mental health training is now a natural part of military service. - Reduction in psychological post-reactions after mission |  |
|  |  |  | - Education of trainers - Education of end users | | - Knowledge of components of the program and support of the program is more widespread (user survey) compared to previous user survey 2021. | |  |  |
|  | **MEASURES**   - User survey aimed at the whole defense to identify: - determinants for implementation, - actual habits, - knowledge about topic - motivational aspects |  | **MEASURES**   - Frequency of training deliveries - Acceptability of program | **MEASURES**   - Fidelity to program delivery (on selected parameters) controlled by military psychologists. | **MEASURES**   - Frequency of training deliveries - Program is enrolled in learning plans at educational institutions. - Follow up user survey. | | |  |
|  |  |  |  |  |  | | **MEASURES**   - QoL after missions (Battery of self-reporting tests) |  |
|  | **ACTIONS**   - Involvement from relevant stakeholders/trainers. Modify program based on feedback. | | | | | |  |  |
|  | **CONTEXT (CULTURE, ENVIRONMENT, TRAINERS, AND END USERS’ CHARACTERISTICS)**  | | | | | | | |
|  | **DARK LOGIC SIDE EFFECTS (potential negatives)** | | | | | | | |
|  | - Lack of “buy in.” Lack of available time - Resistance to the program/trainers | | | | | | | |
|  |  | | | - Ineffective delivery (lack of fidelity) - De-implementation of something else (equally or more important) | | | - Nothing will change | |
