# Supplementary material for: Development of a military mental health training program aiming to promote mental health and operational readiness in the Danish armed forces: an intervention mapping approach
Source: Front Public Health. 2025 Nov 19;13:1676193. doi: 10.3389/fpubh.2025.1676193 (PMC12673934; doi:10.3389/fpubh.2025.1676193)
Supplement: Supplementary file 2 [file Table_2.docx]

Supplementary Material

# Supplementary Online Material

# Initial Logic model (attached in separate file)

# Performance objectives

| **Performance objective #1**  **Increase awareness of the MMT program** | | | |
| --- | --- | --- | --- |
| **Determinant**  **(TDF Domain)** | **Change Objective** | **COM-B** | **BCT(s) Applied** |
| Knowledge | Articulate the purpose and core components of MMT. | Psychological Capability | 3.1 Social support (unspecified);  4.1 Instruction on how to perform the behavior.  5.1 Information about health consequences |
| Information navigation  (Cognitive and interpersonal skills) | Access and navigate the printed leaflets and online materials. | Psychological Capability | 3.1 Social support (unspecified);  4.1 Instruction on how to perform the behavior;  8.1 Behavioral practice/rehearsal |
| Attitudes  (Beliefs about consequences) | Belief MMT enhances operational performance/readiness and overall well-being. | Reflective Motivation | 3.1 Social support (unspecified);  5.6 Information about emotional consequences;  9.1 Credible source |
| Self-efficacy  (Beliefs about capabilities) | Confidence engaging with MMT content and activities. | Reflective Motivation | 1.4 Action planning;  3.1 Social support (unspecified);  15.1 Verbal persuasion about capability |
| Social norms  (Social influences) | Perceive MMT as relevant and endorsed by peers, NCOs, and officers. | Social Opportunity | 3.1 Social support (unspecified);  6.2 Social comparison;  9.1 Credible source |
| Access/logistics (Environmental context and resources) | Informed via flyers, internal notice boards about MMT schedules and procedures and online. | Physical Opportunity | 3.1 Social support (unspecified);  7.1 Prompts/cues;  12.5 Adding objects to the environment |

Performance objective #1 - Spread awareness of the MMT program among soldiers

| **Performance objective #2**  **Integrate MMT into structured military education curricula** | | | |
| --- | --- | --- | --- |
| **Determinant (TDF Domain)** | **Change Objective** | **COM-B** | **BCT(s) Applied** |
| Policy literacy (Knowledge) | Planners understand Defense policies and NATO guidelines supporting MMT integration into curricula. | Psychological Capability | 3.1 Social support (unspecified);  5.1 Information about health consequences; 9.1 Credible source |
| Curriculum alignment  (Skills) | Planners can map MMT modules into course syllabi, assessment frameworks, and accreditation criteria. | Physical Capability | 1.1 Goal setting (behavior);  3.1 Social support (unspecified);  4.1 Instruction on how to perform the behavior |
| Attitudes  (Beliefs about consequences) | Planners believe that institutionalizing MMT improves training and operational effectiveness. | Reflective Motivation | 3.1 Social support (unspecified);  5.6 Information about emotional consequences;  6.3 Information about others’ approval |
| Self-efficacy  (Beliefs about capabilities) | Planners feel equipped to navigate inter-agency approval processes and secure buy-in. | Reflective Motivation | 1.4 Action planning; 3.1 Social support (unspecified); 15.1 Verbal persuasion about capability |
| Organizational norms  (Social influences) | Trainers and course instructors regard MMT as a legitimate, valuable part of professional military education. | Social Opportunity | 3.1 Social support (unspecified);  6.2 Social comparison |
| Structural support (Environmental context and resources) | The Defense allocates time, training slots, and formal structures for MMT implementation. | Physical Opportunity | 3.1 Social support (unspecified);  7.1 Prompts/cues;  12.1 Restructuring the physical environment |

Performance objective #2 - Integrate MMT into structured military education curricula

| **Performance objective #3**  **Increase usage of stress‑management skills in everyday routines** | | | |
| --- | --- | --- | --- |
| **Determinant**  **(TDF Domain)** | **Change Objective** | **COM-B** | **BCT(s) Applied** |
| Knowledge | Recall and distinguish core MMT techniques (e.g., breathing, imagery, self-talk, goal setting). | Psychological Capability | 3.1 Social support (unspecified);  4.1 Instruction on how to perform the behavior;  5.1 Information about health consequences |
| Skill proficiency (Skills) | Competently demonstrate selected stress-management exercises during routine drills | Physical Capability | 3.1 Social support (unspecified);  6.1 Demonstration of the behavior;  8.1 Behavioral practice/rehearsal |
| Attitudes  (Beliefs about consequences) | Value integrating stress-management practices into everyday soldiering tasks. | Reflective Motivation | 3.1 Social support (unspecified);  5.6 Information about emotional consequences;  10.4 Social reward |
| Self-efficacy  (Beliefs about capabilities) | Report confidence in their ability to apply techniques effectively under realistic stressors. | Reflective Motivation | 1.4 Action planning;  3.1 Social support (unspecified);  15.1 Verbal persuasion about capability |
| Social norms  (Social influences) | Observe peers practicing these techniques and feel supported to join in unit activities. | Social Opportunity | 3.1 Social support (unspecified);  6.2 Social comparison |
| Access/time (Environmental context and resources) | Allocate time for skill rehearsal during daily schedules. | Physical Opportunity | 3.1 Social support (unspecified);  7.1 Prompts/cues;  12.5 Adding objects to the environment |

Performance objective #3 – Personnel Increase usage of stress‑management skills in everyday routines

| **Performance objective #4**  **Educate trainers to deliver the MMT program with fidelity** | | | |
| --- | --- | --- | --- |
| **Determinant**  **(TDF Domain)** | **Change Objective** | **COM-B** | **BCT(s) Applied** |
| Knowledge | Explain the theoretical rationale, session flow, and learning objectives of the MMT program. | Psychological Capability | 3.1 Social support (unspecified);  4.1 Instruction on how to perform the behavior;  5.1 Information about health consequences |
| Instructional delivery (Skills) | Facilitate each module accurately, using standard materials and exercises without deviation (fidelity). | Physical Capability | 3.1 Social support (unspecified);  6.1 Demonstration of the behavior;  8.1 Behavioral practice/rehearsal |
| Attitudes  (Beliefs about consequences) | Perceive the MMT program as best practice, relevant, and integral to soldier preparedness. | Reflective Motivation | 3.1 Social support (unspecified);  5.6 Information about emotional consequences; 9.1 Credible source |
| Self-efficacy  (Beliefs about capabilities) | Report confidence in facilitating sessions, managing group dynamics, and responding to participant queries. | Reflective Motivation | 1.4 Action planning;  3.1 Social support (unspecified);  15.1 Verbal persuasion about capability |
| Peer validation (Social influences) | Perceive support from leadership and fellow trainers regarding MMT delivery. | Social Opportunity | 3.1 Social support (unspecified);  6.2 Social comparison |
| Logistical support (Environmental context and resources) | Have protected time, facilities, and materials needed to prepare and deliver MMT sessions. | Physical Opportunity | 3.1 Social support (unspecified);  7.1 Prompts/cues;  12.1 Restructuring the physical environment |

Performance objective #4 - Educate trainers to deliver the MMT program with fidelity
